# The Inhibitory Helix Controls the Intramolecular Conformational Switching of the C-Terminus of STIM1

**DOI:** 10.1371/journal.pone.0074735

**Published:** 2013-09-19

**Authors:** Boyang Cui, Xue Yang, Siwei Li, Zhijie Lin, Zheng Wang, Cheng Dong, Yuequan Shen

**Affiliations:** 1 State Key Laboratory of Medicinal Chemical Biology, Nankai University, Tianjin, China; 2 College of Life Sciences, Nankai University, Tianjin, China; 3 Synergetic Innovation Center of Chemical Science and Engineering, Tianjin, China; Institute of Molecular and Cell Biology, Singapore

## Abstract

Store-operated Ca^2+^ entry (SOCE) is a critical Ca^2+^ signaling pathway in many cell types. After sensing Ca^2+^ store depletion in the endoplasmic reticulum (ER) lumen, STIM1 (STromal Interaction Molecule 1) oligomerizes and then interacts with and activates the Orai1 calcium channel. Our previous research has demonstrated that the inhibitory helix (IH) adjacent to the first coiled-coil region (CC1) of STIM1 may keep the whole C-terminus of STIM1 in an inactive state. However, the specific conformational change of CC1-IH that drives the transition of STIM1 from the resting state to the active state remains elusive. Herein, we report the structural analysis of CC1-IH, which revealed that the entire CC1-IH molecule forms a very long helix. Structural and biochemical analyses indicated that IH, and not the CC1 region, contributes to the oligomerization of STIM1. Small-angle X-ray scattering (SAXS) analysis suggested that the C-terminus of STIM1 including the IH region displays a collapsed conformation, whereas the construct without the IH region has an extended conformation. These two conformations may correspond to the conformational states of the C-terminus of STIM1 before and after activation. Taken together, our results provide direct biochemical evidence that the IH region controls the conformational switching of the C-terminus of STIM1.

## Introduction

Calcium (Ca^2+^) signaling plays a critical role in the regulation of various physiological processes [1]. Ca^2+^ signals can be generated by different pathways, and among these pathways, store-operated calcium entry (SOCE), which is triggered by calcium depletion in the endoplasmic reticulum (ER), is a principal cellular signaling pathway that maintains cellular Ca^2+^ homeostasis [2,3]. Two protein families are involved in this signaling pathway: the ER-localized stromal interaction molecule (STIM) calcium sensors [4,5] and the calcium-release activated calcium (CRAC) channels (consisting of Orai family proteins), which are located in the plasma membrane [6,7,8].

In resting cells, the cytosolic Ca^2+^ concentration is maintained at a relatively stable level due to the Ca^2+^ ATPase pumps SERCA in the ER membrane and PMCA in the plasma membrane [1,2,9]. A series of cellular events are triggered following extracellular ligand binding to phospholipase C (PLC)-coupled receptors on the plasma membrane [10,11,12]. These events finally result in Ca^2+^ release from the ER lumen. Upon sensing the depletion of Ca^2+^ stores in the ER, STIM1 becomes activated and oligomerizes. Next, the STIM1 oligomers rapidly migrate to ER-plasma membrane junctions, where they activate CRAC channels by directly binding to Orai1. Constitutive Ca^2+^ entry is achieved through the opening of CRAC channels, which further elicits intracellular Ca^2+^ signals and replenishes the depleted Ca^2+^ stores in the ER lumen [1,2,3].

Full-length STIM1 consists of 685 amino acids and is a single-pass transmembrane protein that spans the ER membrane, with its N-terminal region (STIM1-N, approximately 22 kDa) located in the ER lumen [2]. STIM1-N is comprised of two EF-hand domains and a sterile α-motif (SAM) [13]. Structural and biochemical studies of STIM1-N have revealed that this region can perform the monomer-to-oligomer transition by itself upon Ca^2+^ release from its first EF-hand, and this conformational change initiates the activation of the entire STIM1 molecule and results in Ca^2+^ entry into the ER lumen [13,14]. The cytoplasmic C-terminus of STIM1 (STIM1-Ccyto, approximately 51 kDa) contains two extensive coiled-coil regions, a Pro/Ser-rich domain and a Lys-rich domain [2,15]. Our recent study of STIM1-Ccyto revealed that it is maintained in an inactive dimeric form in resting cells [16]. The second coiled-coil domain of STIM1-Ccyto (a segment approximately 100 amino acids in length that has been termed the STIM-Orai activating region [SOAR]) mediates the interactions between STIM1 and Orai channels [16,17]. The formation of SOAR dimers can constitutively activate CRAC channels by directly binding to Orai1. However, the SOAR domain must be released from intramolecular inhibition during STIM1 activation [18,19]. Our recent study demonstrated that the inhibitory helix (amino acids 310-337, referred to as IH), which is close to the SOAR dimer, tightly binds to SOAR to prevent its exposure, thus keeping STIM1-Ccyto in an inactive resting state [16]. Upon activation, IH releases the SOAR dimer and then it forms an elongated coiled-coil region, thus allowing SOAR to activate the CRAC channel.

However, a detailed understanding of the STIM1 activation mechanism has not be achieved, largely due to the lack of structural information on human STIM1-Ccyto. The potential contributions of the CC1 region (residues 237-309) and/or IH of STIM1 to the activation of STIM1-Ccyto remain unknown. To better understand the conformational change of CC1 in the presence of the IH region, we determined the crystal structure of a STIM1 construct (amino acids 237-340, hereafter referred to as CC1-IH) and demonstrated the existence of both the inactive and active states of the STIM1 C-terminus in solution.

## Results

### The overall structure of CC1-IH

**Figure 1:**
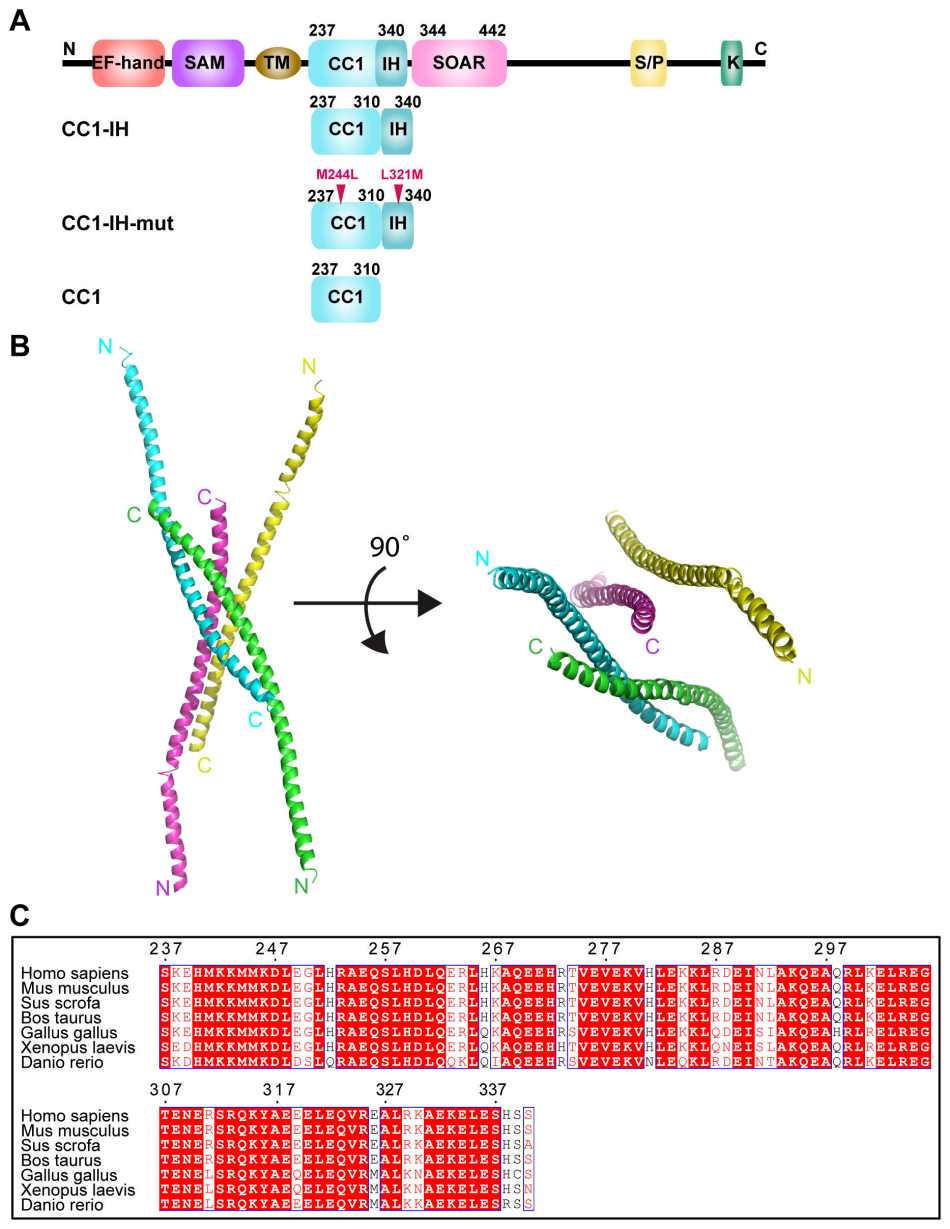
Structure of CC1-IH. (**A**) Schematic layout of the human STIM1 molecule with its coiled-coil regions CC1, IH and SOAR shown in cyan, dark blue and pink, respectively. Below the schematic are the various constructs utilized in our research. (**B**) Left, cartoon of the asymmetric unit, which contains four CC1-IH molecules. In the crystal structure, each CC1-IH molecule forms an elongated α-helix from its N-terminus to its C-terminus. Right, 90° rotation of the image at left. (**C**) Sequence alignment of CC1-IH (amino acids 237-340) from *Homo sapiens*, *Mus musculus*, *Sus scrofa*, *Bos taurus*, *Gallus gallus*, *Xenopus laevis* and *Danio rerio*. Residues that are conserved in all species are highlighted by red boxes; residues that are conserved in all species but one or two are in red. The residue numbers shown in black are based on the human STIM1 sequence.

To obtain a three-dimensional structure for CC1-IH, a series of CC1-IH mutants were utilized in crystallization trials. One CC1-IH mutant (residues 237-340 with two mutations [M244L and L321M], referred to as CC1-IH-mut) yielded high-quality crystals that diffracted to a high resolution (Figure 1A). The structure of CC1-IH-mut was determined using the single-wavelength anomalous dispersion (SAD) method. There are four CC1-IH-mut molecules in one asymmetric unit (Figure 1B), and each molecule consists of an elongated α-helix stretching from its N-terminal end (referred as the head) to its C-terminal end (referred as the tail). The asymmetric unit content resembles four long sticks gathered together (Figure 1B). Furthermore, two of the four CC1-IH-mut molecules have the same spatial orientation, whereas the other two are oriented in the opposite direction. As a result, all the tails of the CC1-IH-mut structure are clustered together, whereas the four heads are spread out. The alignment of CC1-IH sequences from various species shows that the amino acid composition of this segment is highly similar across vertebrate species (Figure 1C), which may help maintain its conserved three-dimensional structure and also indicates that its biological function is evolutionarily conserved.

### Oligomeric state of CC1-IH in solution

**Figure 2:**
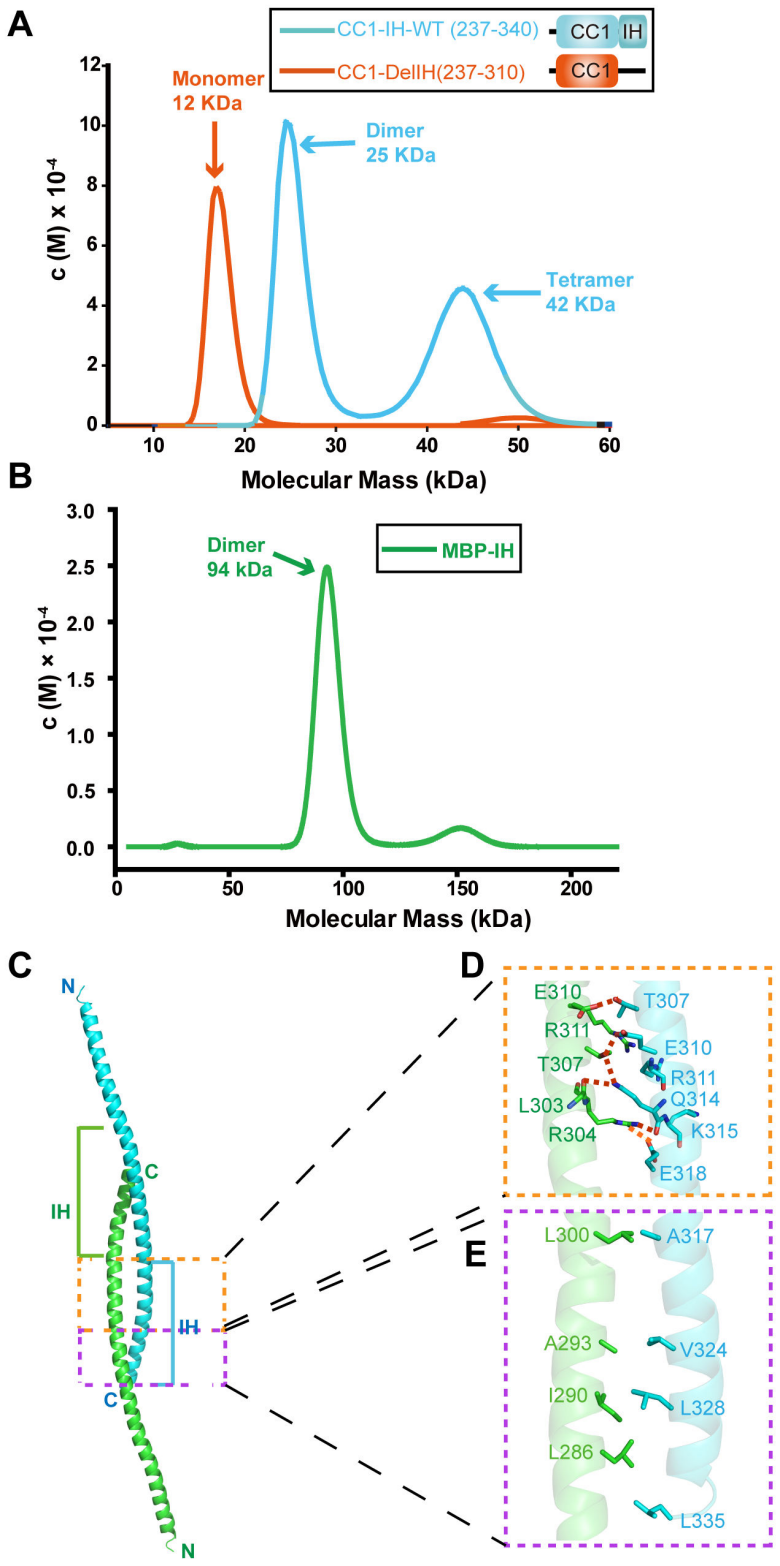
Oligomeric state of CC1-IH in solution. (**A**) Sedimentation velocity analytical ultracentrifugation analysis of wild-type CC1-IH (residues 237-340) and CC1 with the inhibitory helix removed (residues 237-310). The curves shown in blue and orange represent wild-type CC1-IH and CC1, respectively, and their calculated molecular weights are marked in blue and orange, respectively. (**B**) Sedimentation velocity of MBP-tagged IH (residues 310-340). (**C**) A CC1-IH dimer mediated by each inhibitory helix at the extreme C-terminus of each monomer. (**D**, **E**) Detailed view of the interactions within the interface of the CC1-IH dimer. Their interactions are not fully described here because the two helices interacted to form the dimer in a reciprocal manner. Residues are colored based on atom type: carbon is shown in green/cyan (based on the corresponding monomer), oxygen is red, and nitrogen is blue.

To investigate the oligomeric state of CC1-IH in solution, we utilized the analytical ultracentrifugation technique. Wild-type CC1-IH (theoretical mass of 12.5 kD) exists predominantly as a dimer (approximately 25.5 kD) and partially as a tetramer (approximately 42.3 kD) in solution (Figure 2A, blue curve). Structural analysis showed that the oligomerization of CC1-IH may be mediated by the inhibitory helix region of STIM1. The two monomers paired to form the CC1-IH dimer in an antiparallel manner, with a buried surface area of 1,114.5 Å^2^ as calculated by AREAIMOL [20] in the structure of CC1-IH-mut (Figure 2C). Further structural analysis revealed that hydrophobic interactions and some intermolecular forces, such as hydrogen bonds and electrostatic attractions, are present within the interface of the dimer. These interactions mainly take place between the IH of one CC1-IH fragment and a region preceding IH in the other CC1-IH fragment (Figure 2C). Specifically, the side chains of Leu335, Leu328, Val324 and Ala317 on one monomer (Figure 2D, cyan) tend to exhibit hydrophobic interactions with residues Leu286, Ile290, Ala293 and Leu300 on the other monomer (Figure 2E, green). Moreover, several hydrogen bonds are present within the two monomers.

The main-chain oxygen atom of Leu303 and the NH1 atom of Arg304 on the green monomer form hydrogen bonds with the NE2 atom and the main-chain oxygen atom of Gln314 on the cyan monomer, respectively. Similarly, the OG1 atom of Thr307 on the green helix forms hydrogen bonds with both the OE1 atom of Glu310 and the NE2 atom of Gln314 on the cyan monomer. Additionally, the NH2 atom of Arg304 on the green helix is stabilized by the OE1 atom of Glu318 on the cyan monomer via an ionic bond. The contacts within the dimer interface are not fully detailed here because all the interactions described above are reciprocal.

To confirm that IH mediates the oligomerization of CC1-IH, we purified a CC1-only protein. As we expected, CC1 (theoretical mass of 9.0 kDa) exists as a monomer (12.2 kDa) in solution (Figure 2A, orange curve). We then purified an IH-only fragment (theoretical mass of 3.7 kDa), and size-exclusion chromatography showed that IH-only exists as a dimer (approximately 6.6 kDa, Figure S1A). Moreover, we constructed and purified an MBP-IH fusion protein (theoretical mass of 49 kDa). This fusion protein exists as a dimer according to size-exclusion chromatography (approximately 105 kDa, Figure S1B) and analytical ultracentrifugation (approximately 94 kDa, Figure 2B). We also showed that MBP is a monomer (approximately 44 kDa) in solution (Figure S2). Taken together, these results suggest that the IH domain can dimerize, whereas the CC1 region is less likely to self-oligomerize.

### Active and inactive conformational states of STIM1-Ccyto in solution

**Figure 3:**
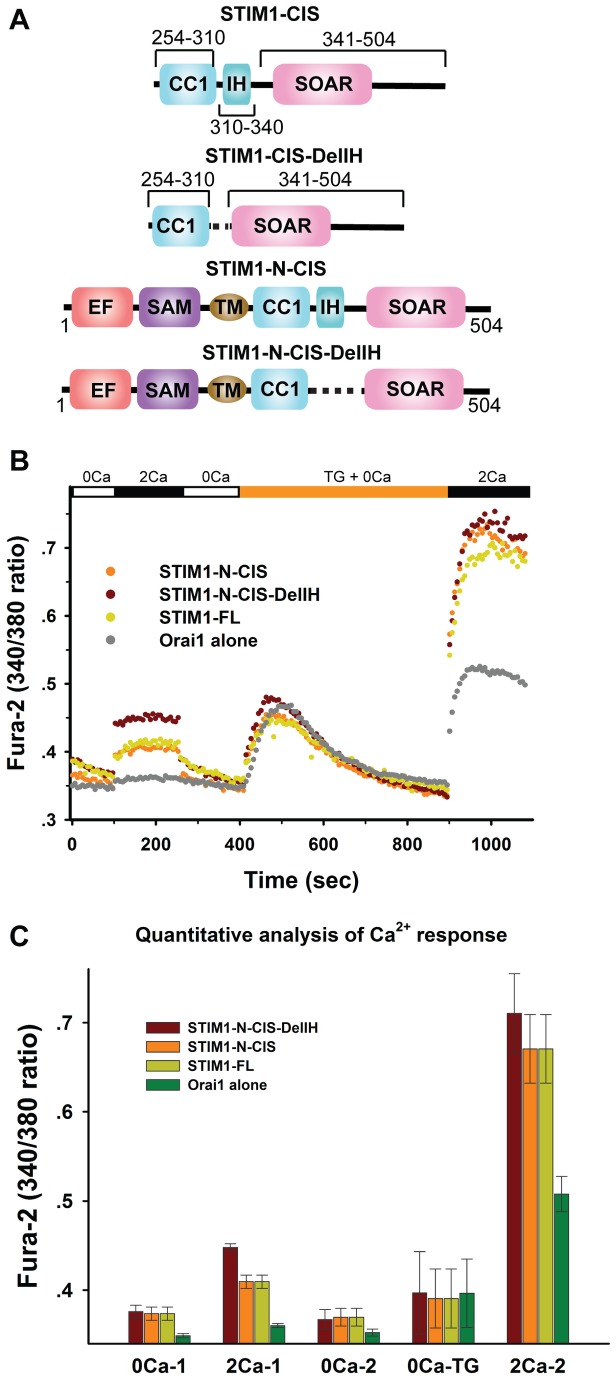
The IH domain keeps SOAR inactive. (**A**) Molecular layout of the simulated inactive and active fragments used in the SAXS analysis and the corresponding fragments used in the intracellular calcium measurement experiments. (**B**) Fura-2 Ca^2+^ measurements of HeLa cells expressing Orai1 alone, Orai1 plus STIM1-N-CIS, Orai1 plus STIM1-N-CIS-DelIH and Orai1 plus STIM1-FL. (**C**) Quantitative analysis of Ca^2+^ fluorescence at different stages.

To further investigate whether the oligomeric state of CC1-IH is related to the conformational change of STIM1-Ccyto from a resting state to an activated state in solution, we selected two STIM1-Ccyto constructs for small-angle X-ray scattering (SAXS) analysis. The two protein samples simulated the resting and activated conformational states of STIM1-Ccyto (especially its coiled-coil regions) in solution. The construct (see Figure 3A) containing residues 254-504 of STIM1-Ccyto (full CC1, IH and SOAR regions included), which we refer to as STIM1-CIS, simulated the resting conformational state of STIM1-Ccyto, whereas the other construct (see Figure 3A) containing residues 254-310 of STIM1-CC1 (IH excluded) and residues 341-504 (SOAR included), which we refer to as STIM1-CIS-DelIH, mimicked the activated conformational state of STIM1-Ccyto. It has been reported that IH is able to maintain the C-terminus of STIM1 in an inactive state *in vivo* based on confocal microscopy and calcium imaging [21]. To confirm different conformational states of STIM1-CIS and STIM1-CIS-DelIH, we performed intracellular Ca^2+^ measurements. It has been reported that STIM1-CIS and STIM1-CIS-DelIH constructs are activated inside cells unless STIM1-N is included [19,21]. Therefore, two additional constructs: STIM1-N-CIS (residues 1-504, Figure 3A) and STIM1-N-CIS-DelIH (residues 1-310 with residues 341-504, Figure 3A) were generated. Orai1 alone, Orai1 plus STIM1-N-CIS-DelIH, Orai1 plus STIM1-N-CIS and Orai1 plus STIM1-FL were transfected into HeLa cells. As shown in Figure 3B and C, the intracellular Ca^2+^ concentrations in the resting state were similar for all constructs when the extracellular Ca^2+^ concentration was 0. The increase in the extracellular Ca^2+^ concentration to 2 mM led to much higher spontaneous Ca^2+^ influx in cells transfected with Orai1 plus STIM1-N-CIS-DelIH than in cells transfected with Orai1, Orai1 plus STIM1-N-CIS or Orai1 plus STIM1-FL. Removing extracellular Ca^2+^ decreased the intracellular Ca^2+^ concentration to the concentration in the resting state for all constructs. After the depletion of Ca^2+^ from ER stores, which led to the maximal activation of the different STIM1 constructs, the Ca^2+^ influxes of cells transfected with Orai1 plus STIM1-N-CIS-DelIH, Orai1 plus STIM1-N-CIS or Orai1 plus STIM1-FL were very similar. These results together indicate that STIM1-N-CIS-DelIH is constitutively active.

**Figure 4:**
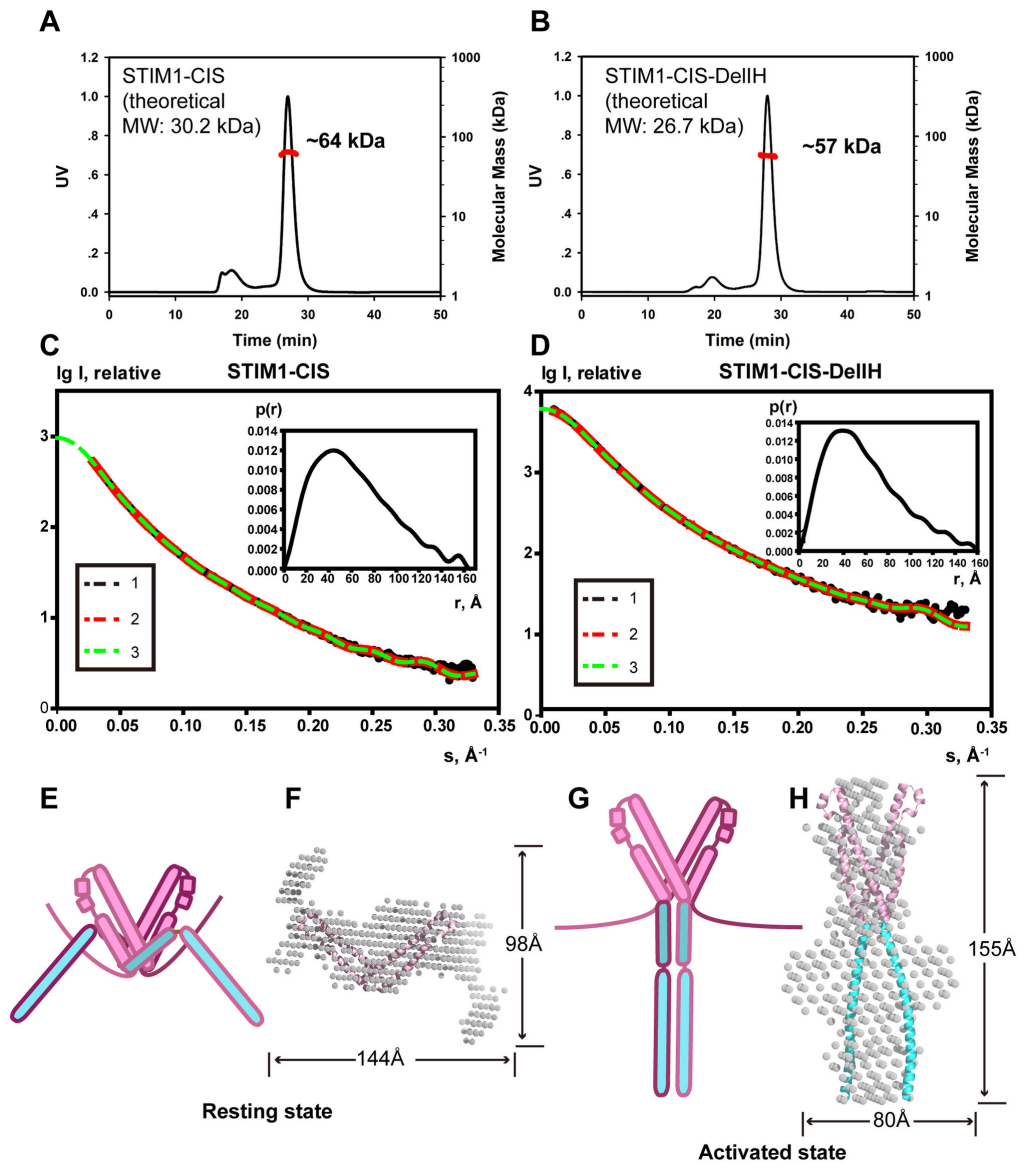
Different conformational states of STIM1-Ccyto in solution as revealed by SAXS. (**A**, **B**) Oligomeric states of STIM1-CIS (**A**) and STIM1-CIS-DelIH (**B**) in solution determined using multi-angle laser scattering. (**C**, **D**) Experimental scattering curves for STIM1-CIS (**C**) and STIM1-CIS-DelIH (**D**) in solution: 1, experimental SAXS curve; 2, scattering patterns calculated from the DAMMIN model; 3, smooth curve back transformed from p(r) and extrapolated to a zero scattering angle for STIM1-CIS. Upper-right, the distance distribution function p(r) computed using the program GNOM. (**E**) Hypothetical molecular shape of STIM1-CIS in solution. STIM1-CIS may adopt a collapsed conformation, which represents the resting state of STIM1-Ccyto. The SOAR and CC1-IH dimers are shown in light pink and cyan, respectively. (**F**) Superimposition of an STIM1-CIS bead model (shown as white dots) developed based on the SAXS data and the known SOAR structure (α-helices shown in light pink). (**G**) Hypothetical molecular shape of STIM1-CIS-DelIH in solution. STIM1-CIS-DelIH may adopt a stretched and elongated conformation when IH is removed (or when IH releases the SOAR dimer), which represents the activated state of STIM1-Ccyto. The SOAR and CC1-IH dimers are shown in light pink and cyan, respectively. (**H**) Superimposition of the STIM1-CIS-DelIH bead model (shown as white dots) developed based on the SAXS data and the known SOAR and CC1 structures (α-helices shown in light pink and cyan, respectively).

STIM1-CIS and STIM1-CIS-DelIH existed as dimers in solution based on the multi-angle laser scattering experiments (Figure 4A and B). The radius of gyration (R_g_) values calculated from the SAXS data using the Guinier and p(r) methods were 49.5 Å and 50.6 Å for STIM1-CIS (Figure 4C and Figure S3A) and 46.4 Å and 46.7 Å for STIM1-CIS-DelIH, respectively (Figure 4D and Figure S3B). The maximum diameter (Dmax) of both proteins was nearly 160 Å (Figure 4C and D, insets). Using low-resolution models restored from the SAXS curves (Figure 4C and D) together with our X-ray crystal structures (CC1-IH and SOAR), we were able to characterize the completely different structural conformations of the two constructs. In the superimposed model of STIM1-CIS (Figure 4E and F), the V-shaped SOAR dimer fits well into the bead envelope calculated from the SAXS data. An extra shadow was present in the bead envelope shown in Figure 4F, suggesting a region that could be occupied by CC1. The overall structural model of STIM1-CIS indicates that STIM1-Ccyto assumes a collapsed conformation in the resting state. In sharp contrast, in the superimposed model of STIM1-CIS-DelIH (Figure 4G and H), the structures of SOAR and CC1-IH successfully fit into the SAXS molecular envelope: the V-shaped SOAR dimer lies in the upper region, and the pair of bar-like CC1-IH structures is located on its lower side. The entire structural model of STIM1-CIS-DelIH adopts a rather stretched and elongated conformation when IH is removed. This structure may help explain the activated conformational state of STIM1-Ccyto after IH releases the SOAR dimer. Taken together, the structural data from SAXS verified our hypothesis that STIM1-Ccyto assumes completely different conformational states before and after activation, and this conformational change is most likely induced by the release of the inhibitory helix from SOAR.

## Discussion

In cells, SOCE is an essential signaling pathway that elicits intracellular Ca^2+^ signals and maintains a normal Ca^2+^ level in the ER [1,2]. The induction of Ca^2+^ influx through the CRAC channel, which replenishes the depleted Ca^2+^ stores in the ER lumen, is achieved by the direct binding of STIM1 oligomers to Orai1 [4,5]. Our previous research has long been focused on understanding the activation mechanism of the C-terminus of STIM1, especially its coiled-coil regions. In our proposed activation model for STIM1 [16], we demonstrated that SOAR dimerization is necessary for the binding of STIM1 with Orai1, and we also determined that the inhibitory helix located close to SOAR tightly interacts with the SOAR dimer to prevent its exposure. The shielding of the SOAR dimer maintains STIM1-Ccyto in the inactive resting state, consistent with other reports that STIM1 is autoinhibited in the resting state [18,19]. However, direct biochemical evidence supporting the resting-to-active switch model is lacking.

Our current study revealed that CC1-IH exists as a long helix and that the oligomerization of CC1-IH depends on the IH domain. Because the SOAR domain was absent from our CC1-IH structure, the observed IH assembly may not be an accurate reflection of how IH is positioned within the entire STIM1 molecule. Docking studies [22] revealed that IH-only can form a parallel coiled-coil dimer (Figure S4). Multiple mutations (E322A-Q323L-E326L-K330L-E332L-K333A) within IH are required to disrupt its amphipathic nature; thus, mutant STIM1 is inactive based on confocal microscopy and intracellular calcium imaging [23]. Furthermore, the structure of STIM1 in solution revealed that its C-terminus assumes a collapsed/extended conformational state with/without IH. Taken together, these data suggest that the IH region plays an important role in controlling the conformational change of the STIM1 C-terminus. Therefore, for the first time, we have provided direct biochemical evidence to demonstrate that IH functions as a switch to inhibit and activate the C-terminus of STIM1 by capturing and releasing the SOAR domain. Our results improve our understanding of the molecular mechanism of SOCE, which is an important cellular process.

## Materials and Methods

### Cloning and expression

The coding sequence of CC1-IH (amino acids 237-340) from human STIM1 was cloned into a modified pET-32a (+) vector (Novagen) in which the Trx-tag, the S-tag and the thrombin recognition site has been replaced with PreScission protease-cleavable segments. *Escherichia coli* BL21(DE3) Codon-Plus cells harboring the recombinant expression plasmid were incubated at 310 K in LB medium containing 50 µg/ml ampicillin and were induced at an OD_600_ of 0.6 with isopropyl-β-D-thiogalactopyranoside (IPTG) at a final concentration of 0.2 mM at 298 K for 4-5 hours. The cells were isolated by centrifugation at 5,000 rpm for 15 minutes. For the expression of Se-Met derivative proteins, two mutations (M244L and L321M) were introduced to improve the quality of the Se-Met crystals. The M244L mutant yielded the best crystal growth for the Se-substituted proteins, and the L321M mutant was used to enhance the anomalous signals for structure determination. All point mutations were created using a standard PCR-based mutagenesis method and were confirmed by DNA sequencing. The recombinant plasmid was transformed into a methionine auxotrophic *E. coli* strain (B834). The cells were cultured and harvested following the same protocol as that used for the wild-type protein with the exception that minimal medium was used to express the recombinant protein.

The cloning and expression of the two constructs used in the SAXS analysis, STIM1-CIS (residues 254-504) and STIM1-CIS-DelIH (residues 254-310 plus residues 341-504), were performed following the protocol described above for CC1-IH. Additionally, the STIM1-CIS-DelIH construct (residues 254-310 plus residues 341-504) was created using a standard PCR-based mutagenesis method to join the two fragments together, and the sequence was confirmed by DNA sequencing.

Regarding the MBP-IH construct, the coding sequence of CC1-IH was first cloned into a modified pET-MBP vector (Novagen) in which the Trx-tag, the S-tag and the thrombin recognition site had been replaced by the MBP-tag plus PreScission protease-cleavable segments. The construct was then obtained using standard PCR-based loop-out mutagenesis with the MBP-CC1-IH recombinant plasmid as a template to remove the entire CC1 gene fragment. The construct was confirmed by DNA sequencing. Subsequent expression work was performed following the same protocol as that described above for CC1-IH.

### Protein purification

The cell pellets were resuspended in 20 mM Tris-HCl (pH 8.0), 200 mM NaCl and 0.1 mM phenylmethylsulfonyl fluoride (PMSF). The supernatant was loaded onto a Ni-NTA column pre-treated with equilibrium buffer (20 mM Tris-HCl [pH 8.0], 200 mM NaCl, 0.1 mM PMSF) and eluted with 20 mM Tris-HCl (pH 8.0), 200 mM NaCl, 200 mM imidazole (pH 8.0) and 0.1 mM PMSF. The 6x His-tagged fusion protein was digested overnight with PreScission protease at a molar ratio of 400:1 (fusion protein:protease) to remove the 6x His tag. The mixture was then diluted with 20 mM Tris-HCl (pH 8.0) to a final NaCl concentration of less than 50 mM and loaded onto a Q-Sepharose anion-exchange column (GE Healthcare). The target protein was eluted with an NaCl gradient (50-250 mM). The eluted fractions were pooled and concentrated. Further purification was performed by gel filtration using a HighLoad 26/60 Superdex-200 size-exclusion column (GE Healthcare) in 20 mM Tris-HCl (pH 8.0), 200 mM NaCl and 1 mM EDTA. The protein purity was determined by SDS-PAGE, and the purified protein was concentrated to 30 mg/ml using a Centricon (Millipore) for crystallization. The Se-Met derivative of CC1-IH-mut and the other constructs (CC1-IH-mut, CC1, STIM1-CIS and STIM1-CIS-DelIH) were purified following the same protocol as that used for the wild-type protein.

The MBP-IH fusion protein was purified following the same protocol as that used for CC1-IH except that the supernatant was first loaded onto an MBPTrap column (GE Healthcare) instead of a Ni-NTA column. In addition, the MBP tag was not cut off and was instead maintained throughout the entire purification process. The IH-only protein was obtained by overnight PreScission protease digestion of the MBP-IH fusion protein at a molar ratio of 400:1 and was finally purified using a Superdex 200 size-exclusion column (GE Healthcare).

### Crystallization and data collection

Crystals were grown using the hanging-drop vapor-diffusion technique. A 1-µl aliquot of the protein solution (30 mg/ml in 20 mM Tris-HCl [pH 8.0], 200 mM NaCl and 1 mM EDTA) was mixed with 1 µl of reservoir solution consisting of 0.1 M NaAc (pH 4.5), 24% PEGMME 2000, 0.02 M NiCl_2_.6H_2_O, 0.02 M CdCl_2_. 6H_2_O and 0.02 M MgCl_2_. 6H_2_O and equilibrated against 400 µl of reservoir buffer. Crystals were grown for 4-5 days at 293 K and frozen in a cryoprotectant consisting of the reservoir solution supplemented with 4 M lithium formate. The Se-Met crystals were produced in the same manner as the wild-type crystals except that the cryoprotectant consisted of reservoir solution supplemented with 25% ethylene glycol.

All the data were collected on station BL17U1 of the Shanghai Synchrotron Radiation Facility (SSRF) and processed using HKL2000 software [24]. Single-wavelength anomalous data were collected for Se-Met-substituted crystals at the peak wavelength for Se.

### Structure determination and refinement

The CC1-IH crystal structure was determined by single-wavelength anomalous dispersion. The program HKL2MAP [25] was used to search for three Se sites, and the initial phases were then calculated using PHENIX software [26]. The model was built manually using the program COOT [27]. After the initial main-chain model was built, the wild-type data were applied to perform an iterative refinement to assign all side chains using COOT. After several refinement cycles, the orientations of the amino acid side chains, bound water molecules and metal atoms were modeled on the basis of 2***F***_obs_ – ***F***_calc_ and ***F***_obs_ – ***F***_calc_ difference Fourier maps. The final structure had an ***R***_crystal_ value of 22.96% and an ***R***_free_ value of 28.74%. Detailed data collection and refinement statistics are summarized in Table 1.

**Table 1:** Data collection and refinement statistics for CC1-IH-mut structure.

**Crystal name**	**CC1-IH-mut**	**Se-Met CC1-IH-mut**
*A. Data collection*		
Space group	***P***3_2_2_1_	***P***3_2_2_1_
Unit cell (Å)	***a***=***b***=101.9, ***c***=104.6, **β**=120.0	***a***=***b***=101.5, ***c***=104.5, **β**=120.0
Wavelength (Å)	0.9792	0.9792
Resolution range (Å)	50.0-2.60 (2.69-2.60)^a^	50.0-3.60 (3.73-3.60)^a^
No. of unique reflections	19,528	13,966
Redundancy	10.5 (11.0)^a^	11.2 (11.6)^a^
***R***_sym_ (%)^b^	7.3 (68.4)^a^	9.6 (39.9)^a^
***I/σ***	31.6 (4.5)^a^	39.9 (8.8)^a^
Completeness (%)	99.5(100)^a^	99.4 (100)^a^
Figure of Merit		0.328
*B. Refinement*		
Resolution range (Å)	33.71-2.60	
***R***_crystal_ (%)^c^	22.96	
***R***_free_ (%)^d^	28.74	
RMSD_bond_(Å)	0.009	
RMSD_angle_(°)	1.107	
Number of protein atoms	2,855	
Solvent atoms	62	
Metal atoms	3	
Residues in (%) most favored	95.1	
Additional allowed	4.9	
Generously allowed	0	
Disallowed	0	
Average B factor (Å^2^) of		
Chain A	69.67	
Chain B	68.53	
Chain C	54.48	
Chain D	66.35	
Metal atoms	68.17	
solvent	50.49	

^a^ the highest resolution shell; ^b $R_{sym}=\sum_{j}\left|\langle I\rangle -I_{j}\right|/\sum \langle I\rangle$^

^c^ $R_{crystal}=\sum_{hkl}\left|F_{obs}-F_{calc}\right|/\sum_{hkl}F_{obs}$

^d^ ***R***_free_, calculated the same as ***R***_crystal_, but from a test set containing 5% of data excluded from the refinement calculation.

### Analytical ultracentrifugation

The sedimentation velocity (SV) was determined using a Beckman/Coulter XL-I analytical ultracentrifuge. The experiments were conducted at 42,000 rpm and 4°C using interference detection and double-sector cells loaded at approximately 6 mg/ml for CC1-IH (residues 237-340), 1.57 mg/ml for CC1 (residues 237-310) and 1.2 mg/ml for the MBP-IH fusion protein. The protein sample buffer contained 20 mM Tris-HCl (pH 8.0), 200 mM NaCl and 1 mM EDTA. The data were analyzed using a self-associating multimerization model with the manufacturer’s software [28].

### Size-exclusion chromatography

A HiLoad 26/60 Superdex 200 size-exclusion column (GE Healthcare) and a Superdex 200 size-exclusion column were both used to evaluate the oligomerization state. The columns were pre-equilibrated with 20 mM Tris-HCl (pH 8.0), 200 mM NaCl and 1 mM EDTA and calibrated with protein standards. The MBP-IH fusion protein and the IH-only protein were injected into the columns and eluted at a flow rate of 2.0 and 0.3 ml/min, respectively.

### Multi-angle laser scattering

Multi-angle light scattering (MALS) were performed in-line with size-exclusion chromatography using a eighteen-angle DAWN HELLOS II instrument equipped with an Optilab rEX Refractive Index Detector (Wyatt Technology). Two hundred microliters of 200 µM protein samples were injected into a Superdex 200 10/300 GL column (GE Healthcare) equilibrated with buffer containing 20 mM Tris-HCl (pH 8.0), 200 mM NaCl, 1 mM DTT and 1 mM EDTA at a flow rate of 0.5 ml/min. The protein concentrations were calculated from the absorbance at 280 nm, and the light scattering data were collected at 663 nm. The molecular weight was calculated using ASTRA (Wyatt Technology).

### SAXS experiments and data analysis

The small-angle X-ray scattering (SAXS) experiments were performed on beamline X33 of the European Molecular Biology Laboratory (EMBL) (Hamburg outstation) at the DORIS III storage ring of the Deutsches Elektronen Synchrotron (DESY) [29]. The STIM1-CIS and STIM1-CIS-DelIH samples were stored in a buffer containing 20 mM Tris (pH 8.0), 200 mM NaCl, 1 mM DTT and 1 mM EDTA. The scattering curves of the two samples (each over a concentration series of 2.5, 5.0 and 10 mg/ml) were recorded over the range of the momentum transfer 0.07 < s < 5.5 nm_1, where s = (4πsinθ)/λ, 2θ is the scattering angle and the wavelength of the X-rays (λ) = 0.15 nm. The measurements were performed in a vacuum cuvette with different exposure times (ranging from 30 sec to 2 min) to reduce parasitic scattering. The experimental scattering profiles of the samples were then corrected for background scattering by the solvent and processed using the program PRIMUS [30] to further extrapolate to zero concentration. The distance distribution function p(r) was calculated using the program GNOM [31], which determines the maximum size of the protein in solution. Its output file was then used for *ab initio* shape reconstruction using the program DAMMIN [32]. The chi-square value for the discrepancy relative to the raw data was 2.466 for STIM1-CIS and 2.665 for STIM1-CIS-DelIH. The constructed low-resolution bead envelope was then overlaid with the known CC1-IH and SOAR structures using the program PYMOL (DeLano Scientific, San Carlos, CA, USA) to construct a final superimposed model.

### Intracellular calcium measurement

HeLa cells (ATCC) were cultured in Dulbecco’s modified Eagle’s medium (DMEM, Sigma-Aldrich) supplemented with 10% v/v fetal bovine serum (FBS, HyClone, Thermo Fisher Scientific). The cells were maintained in a 95% air and 5% CO_2_ environment at 37°C. Gene fragments corresponding to full-length human STIM1 (STIM1-FL) were cloned into the pEYFP-N1 vector (Clontech). The mutants (STIM1-N-CIS and STIM1-N-CIS-DelIH) were made using a standard PCR-based mutagenesis method and were confirmed by DNA sequencing. Human Orai1 was cloned into the pECFP-N1 vector (Clontech).

HeLa cells were plated onto a 24-well plate (Costar) and transfected at 90% confluency with 0.7 µg of DNA per well using Lipofectamine 2000. Transfected cells were loaded with 1 µM Fura-2 AM (Sigma-Aldrich) in DMEM with 10% FBS for 30 min at 37°C. After loading, the cells were rinsed in 2 mM Ca^2+^ Ringer’s solution containing 145 mM NaCl, 1 mM MgCl_2_, 4.5 mM KCl, 10 mM glucose, 20 mM HEPES (pH 7.4) and 2 mM CaCl_2_ for 20 min. Excitation at 340 nm and 380 nm and emission at 508 nm were monitored using a Synergy4 spectrometer (BioTek). Data representing the relative intracellular Ca^2+^ concentrations are reported as 340/380 ratios.

### Accession number

The atomic coordinates and structure factors for the structure of CC1-IH have been deposited in the Protein Data Bank with accession code 4IOZ.

## Supporting Information

Figure S1 **Oligomerization state of purified IH-only and IH-MBP fusion proteins.** The IH-only protein (**A**) and the MBP-IH fusion protein (**B**) were loaded onto a HiLoad Superdex 200 column. A protein marker was used to calibrate the column. The molecular masses are shown at the top of the elution profile and are marked with black arrows. The molecular masses of the proteins were calculated based on a standard curve generated from the retention volumes of the protein maker. Inset, fractions from each peak were loaded onto a 15% SDS-PAGE gel and stained with Coomassie blue.
(TIF)

Figure S2 **Sedimentation velocity analytical ultracentrifugation of MBP.** The experimental molecular weight of MBP was approximately 44 kDa (predicted molecular weight, 43 kDa), indicating that the MBP protein is a monomer in solution.
(TIF)

Figure S3 **Low-q Guinier plot for STIM1 mutants.** (**A**) Low-q Guinier plot for the model of STIM1-CIS. The radius of gyration (Rg) calculated by the Guinier method is 49.5. (**B**) Low-q Guinier plot for the model of STIM1-CIS-DelIH. The radius of gyration (Rg) calculated by the Guinier method is 46.4.
(TIF)

Figure S4 **Protein docking model of two IH helices created with the ZDOCK program.** (**A**) Cartoon representation of the best docking complex model of a potential IH dimer with parallel alignment. (**B**) The potential IH dimer is shown as a stick model.
(TIF)
