# Supplementary figures and images for: The Inhibitory Helix Controls the Intramolecular Conformational Switching of the C-Terminus of STIM1
Source: PLoS One. 2013 Sep 19;8(9):e74735. doi: 10.1371/journal.pone.0074735 (PMC3777995; doi:10.1371/journal.pone.0074735)

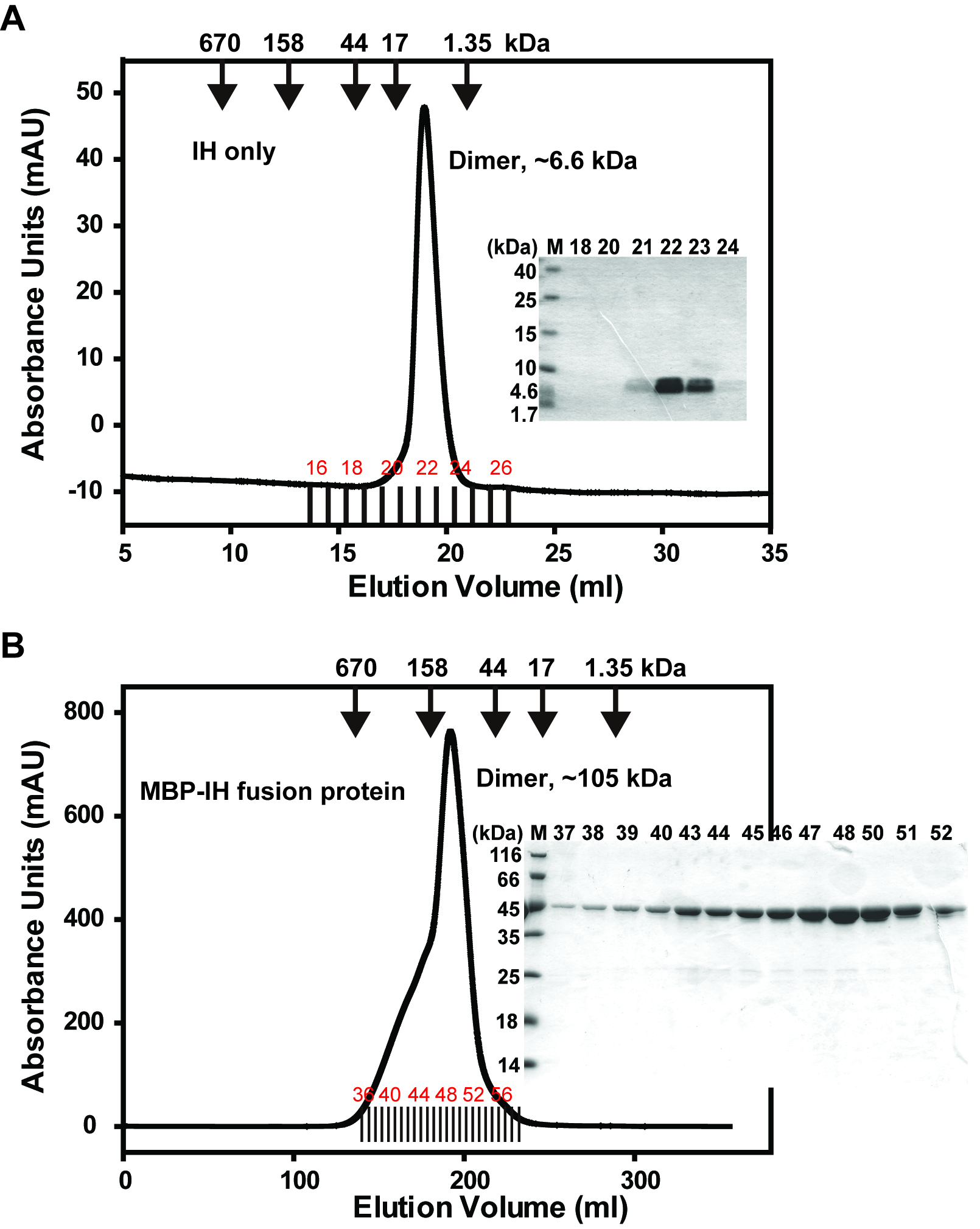

Supplement: Figure S1 — Oligomerization state of purified IH-only and IH-MBP fusion proteins. The IH-only protein (A) and the MBP-IH fusion protein (B) were loaded onto a HiLoad Superdex 200 column. A protein marker was used to calibrate the column. The molecular masses are shown at the top of the elution profile and are marked with black arrows. The molecular masses of the proteins were calculated based on a standard curve generated from the retention volumes of the protein maker. Inset, fractions from each peak were loaded onto a 15% SDS-PAGE gel and stained with Coomassie blue. (TIF) [file pone.0074735.s001.tif]

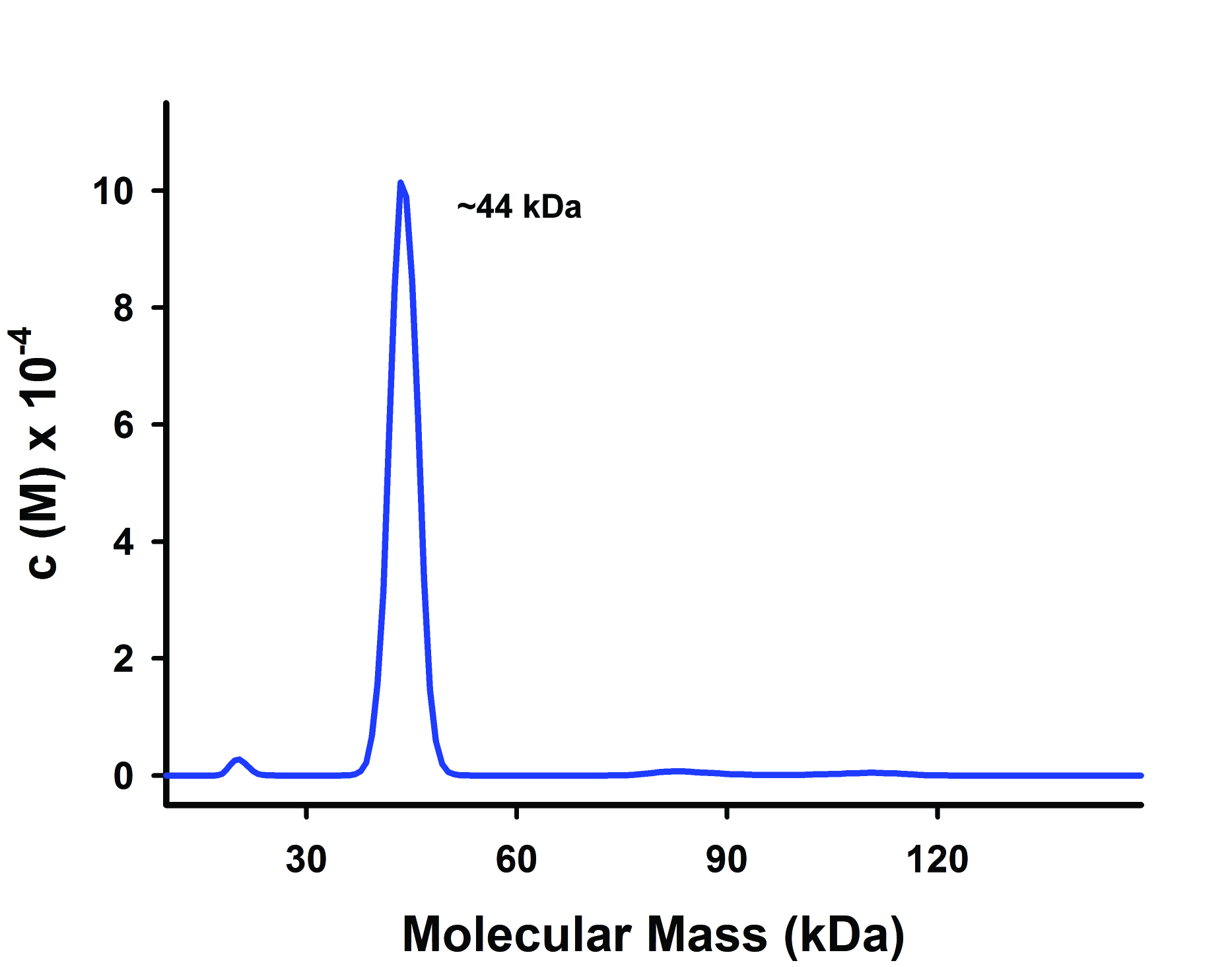

Supplement: Figure S2 — Sedimentation velocity analytical ultracentrifugation of MBP. The experimental molecular weight of MBP was approximately 44 kDa (predicted molecular weight, 43 kDa), indicating that the MBP protein is a monomer in solution. (TIF) [file pone.0074735.s002.tif]

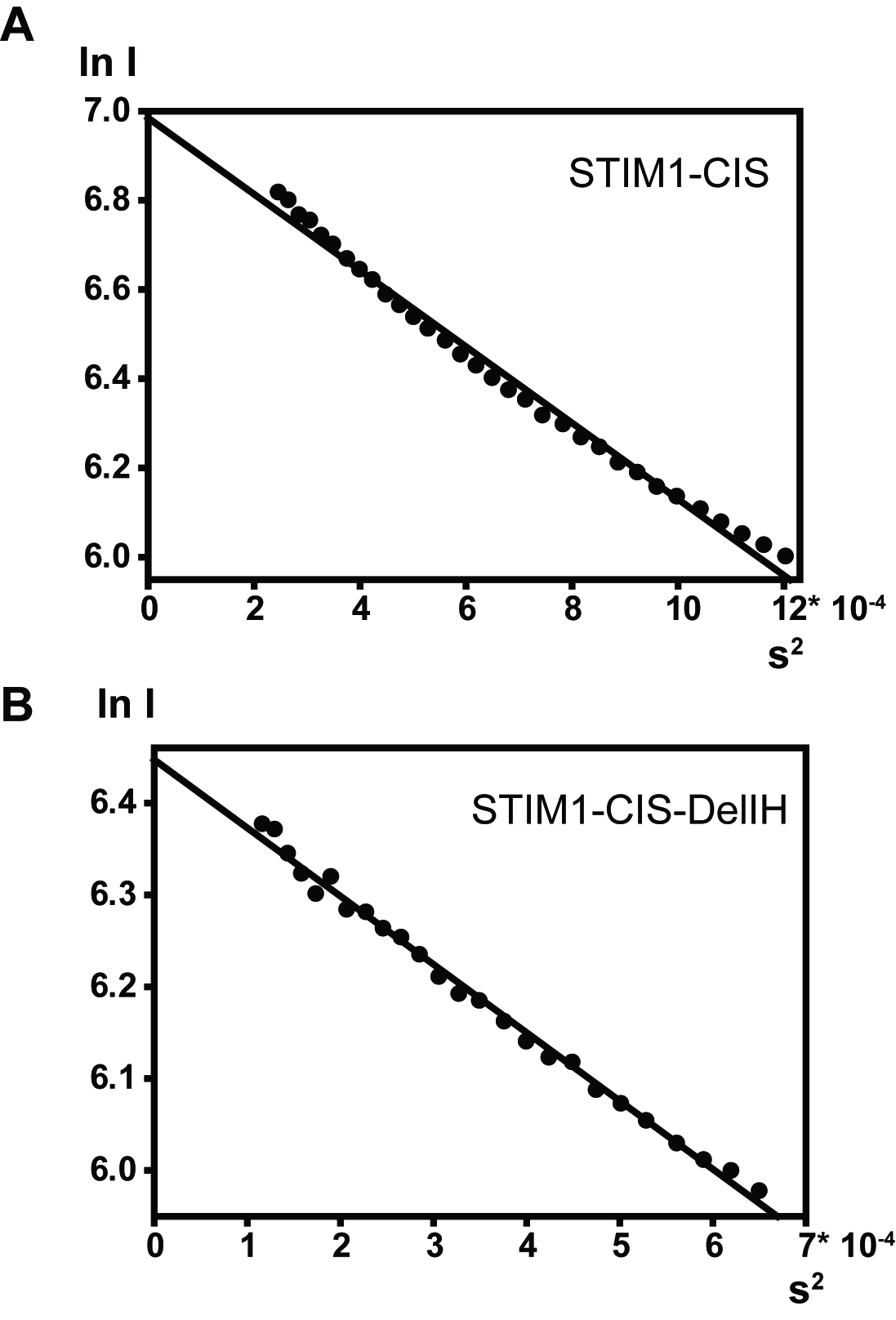

Supplement: Figure S3 — Low-q Guinier plot for STIM1 mutants. (A) Low-q Guinier plot for the model of STIM1-CIS. The radius of gyration (Rg) calculated by the Guinier method is 49.5. (B) Low-q Guinier plot for the model of STIM1-CIS-DelIH. The radius of gyration (Rg) calculated by the Guinier method is 46.4. (TIF) [file pone.0074735.s003.tif]

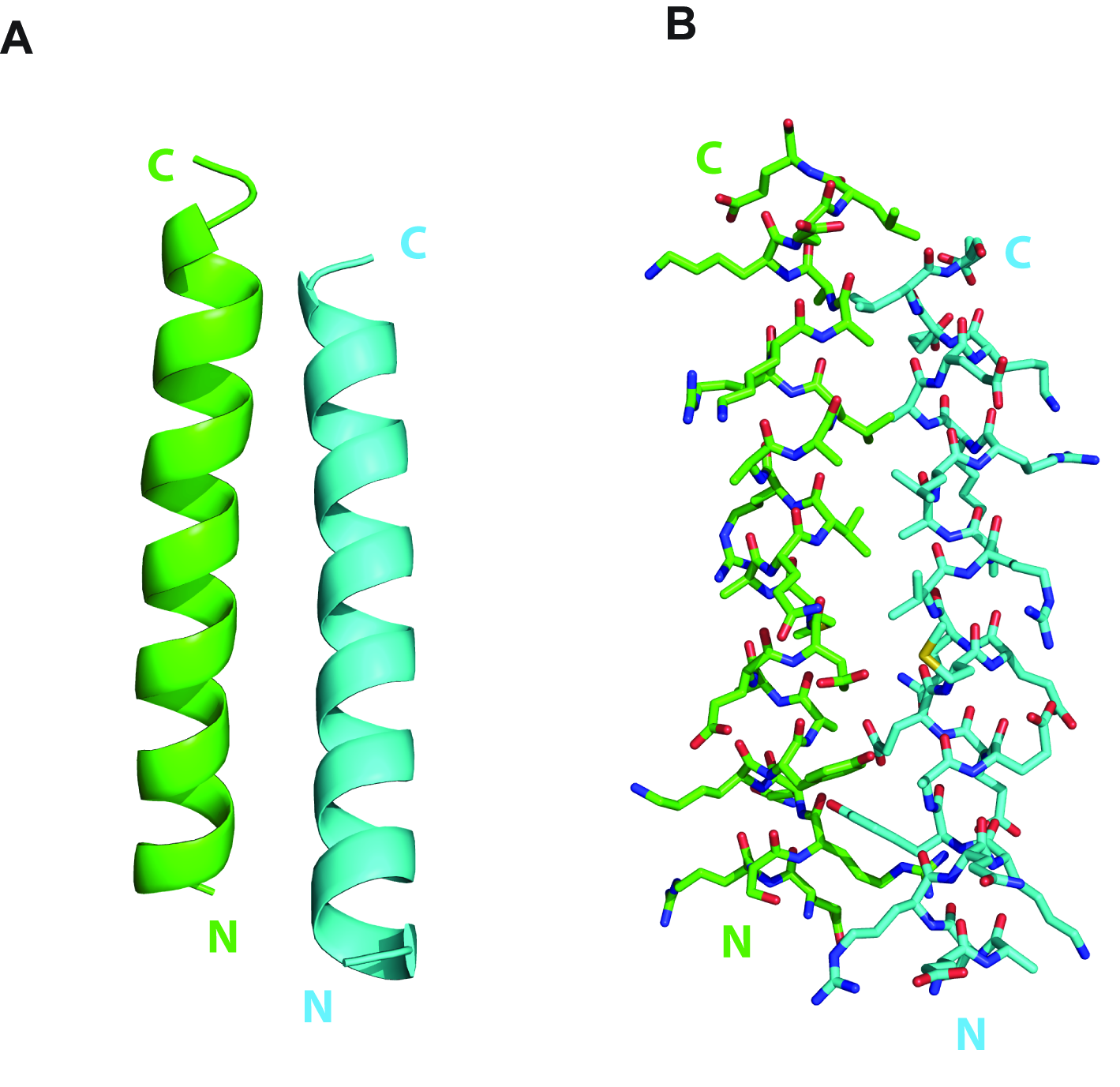

Supplement: Figure S4 — Protein docking model of two IH helices created with the ZDOCK program. (A) Cartoon representation of the best docking complex model of a potential IH dimer with parallel alignment. (B) The potential IH dimer is shown as a stick model. (TIF) [file pone.0074735.s004.tif]
